# Identification of tRNA-derived ncRNAs in TCGA and NCI-60 panel cell lines and development of the public database tRFexplorer

**DOI:** 10.1093/database/baz115

**Published:** 2019-11-18

**Authors:** Alessandro La Ferlita, Salvatore Alaimo, Dario Veneziano, Giovanni Nigita, Veronica Balatti, Carlo M Croce, Alfredo Ferro, Alfredo Pulvirenti

**Affiliations:** 1 Department of Physics and Astronomy, University of Catania, Catania, Italy; 2 Department of Clinical and Experimental Medicine, University of Catania, Catania, Italy; 3 Department of Cancer Biology and Genetics, Comprehensive Cancer Center, The Ohio State University, Columbus, OH

## Abstract

Next-generation sequencing is increasing our understanding and knowledge of non-coding RNAs (ncRNAs), elucidating their roles in molecular mechanisms and processes such as cell growth and development. Within such a class, tRNA-derived ncRNAs have been recently associated with gene expression regulation in cancer progression. In this paper, we characterize, for the first time, tRNA-derived ncRNAs in NCI-60. Furthermore, we assess their expression profile in The Cancer Genome Atlas (TCGA). Our comprehensive analysis allowed us to report 322 distinct tRNA-derived ncRNAs in NCI-60, categorized in tRNA-derived fragments (11 tRF-5s, 55 tRF-3s), tRNA-derived small RNAs (107 tsRNAs) and tRNA 5′ leader RNAs (149 sequences identified). In TCGA, we were able to identify 232 distinct tRNA-derived ncRNAs categorized in 53 tRF-5s, 58 tRF-3s, 63 tsRNAs and 58 5′ leader RNAs. This latter group represents an additional evidence of tRNA-derived ncRNAs originating from the 5′ leader region of precursor tRNA. We developed a public database, tRFexplorer, which provides users with the expression profile of each tRNA-derived ncRNAs in every cell line in NCI-60 as well as for each TCGA tumor type. Moreover, the system allows us to perform differential expression analyses of such fragments in TCGA, as well as correlation analyses of tRNA-derived ncRNAs expression in TCGA and NCI-60 with gene and miRNA expression in TCGA samples, in association with all omics and compound activities data available on CellMiner. Hence, the tool provides an important opportunity to investigate their potential biological roles in absence of any direct experimental evidence.

Database URL: https://trfexplorer.cloud/

## Introduction

With the advent of next-generation sequencing technologies, the number of characterized ncRNA classes in eukaryotic cells has dramatically increased (1–3). Recently, tRNA-derived non-coding RNAs (ncRNAs), a novel heterogeneous class of ncRNAs originating from tRNA processing, have been characterized. Indeed, it has been shown that tRNA-derived ncRNAs are not mere byproducts of random tRNA cleavage, rather they may actively play roles in several biological phenomena, such as ribosome biogenesis, retrotransposition, virus infections, apoptosis and cancer pathogenesis (4–13). Furthermore, some classes of tRNA-derived ncRNAs have been shown to bind AGO and PIWI proteins, potentially acting as post- or pre-transcriptional regulators of gene expression (9, 14).

Accumulating evidence also suggests the presence of functional tRNA-derived ncRNAs in human biological fluids, such as urine and serum from cancer patients (15–19).

tRNA biogenesis begins with the transcription of tRNA genes by RNA polymerase III leading to ‘precursor tRNA’ (pre-tRNA). Such molecules undergo a maturation process inside the nucleus, where 5′ leader and 3′ trailer sequences are cleaved by ribonuclease P (RNase P) and ribonuclease Z (RNase Z), respectively (15, 20–25). In the last few years, several kinds of tRNA-derived ncRNAs have been discovered. However, a unique classification is still missing. A common grouping of such molecules is based on the location they originate from within the tRNA gene. tRNA-derived ncRNAs can, therefore, be divided into two main classes: (i) tsRNAs, which derive from pre-tRNA and (ii) stress-induced tRNA fragments (tiRNAs), together with tRFs, which derive from mature tRNA (13).

tsRNA are produced inside the nucleus and result from the cleavage of the pre-tRNAs 3′ trailer sequence by RNases Z. They usually begin after the 3′-end of mature tRNAs and are characterized by a polyuracil sequence at their 3′-ends (13).

tiRNAs, which have a length of ~28–36 nt, are produced in the cytoplasm via specific cleavage of the anticodon loop of mature tRNAs by Rny1p and angiogenin (ANG) in yeast and mammalians cells, respectively (15, 26, 27). This class is comprised of 5’-tiRNA and 3’-tiRNA, in reference to the 5′ or 3′ half of the mature tRNA they derive from, respectively (15).

tRFs, ranging from 14 to 30 nt in length, are derived from mature tRNA (15, 23, 28). Three types of tRFs have been discovered to date: (i) tRF-5s; (ii) tRF-3s; and (iii) i-tRFs (29, 30). tRF-5s are generated in the cytoplasm by Dicer-mediated cleavage of the mature tRNA D-loop (29, 31). tRF-3s are produced in the cytoplasm via cleavage of the T-loop in mature tRNAs operated by Dicer, ANG and other members of the RNase A superfamily. They are fragments originating from mature tRNA 3′-ends, and include the final CCA sequence (28, 29, 32). Finally, i-tRFs are enriched within the internal regions of mature tRNAs, usually straddling the anticodon region (29, 33). It is important to highlight that in literature and in some databases, tsRNAs (which derive from 3′ trailer sequence of pre-tRNAs) are also termed tRF-1s (30, 34, 35).

Additionally, a recently investigated group of tRFs, namely 5′ leader-exon tRFs, has been described in a study associating them with the loss of spinal motor neurons in CLP1-kinase dead mice (36). These fragments span from the beginning of the 5′ end of pre-tRNAs to the 5′ end of mature tRNAs and are produced inside the nucleus. However, their biogenesis and function remain still unknown, and consequently, this class has not currently been completely characterized (36).

Our study aims at the identification of tRNA-derived ncRNAs in the National Cancer Institute 60 (NCI-60) cell lines and The Cancer Genome Atlas (TCGA) samples. This has been done through the development of a custom bioinformatics pipeline for the identification of tsRNAs (also termed tRF-1), tRFs (tRF-5s and tRF-3s) and 5′ leader RNAs in small non-coding RNA-seq (sncRNA-seq data).

NCI-60 is a panel of 60 human cancer cell lines derived from nine different cancer types (leukemia, colon, lung, central nervous system, renal, melanoma, ovarian, breast and prostate) (37–39), while TCGA is a collection of samples covering 33 different tumor types with more than 11 000 cancer patients.

We collected all the profiling results in an intuitive, publicly available database, tRFexplorer (https://trfexplorer.cloud/). Our database allows users to search for tRNA-derived ncRNAs, visualize their expression profiles in both NCI-60 cell lines and TCGA patient cohorts, apply differential expression (DE) analysis on TCGA samples and correlate tRNA-derived ncRNA expression profiles with several covariates, such as NCI-60 omics data, TCGA mRNA and miRNA expressions, and TCGA patients survival data, in order to better aid in the identification of mechanisms in which such molecules might be involved in.

## Material and methods

### tRNA-derived ncRNAs identification pipeline

The identification of tsRNAs, 5′ leader RNAs and tRFs in sncRNA-seq datasets is a complex process, since such small fragments may be mapped to multiple DNA regions. For this purpose, we implemented a conservative pipeline to get an accurate estimation of tsRNAs, 5′ leader RNAs and tRF expression. First, we assembled a custom annotation of the reference human genome (hg19) containing only known tsRNAs and tRFs. We included all tRF-5s, tRF-3s and tRF-1s from tRFdb (http://genome.bioch.virginia.edu/trfdb/) (34), all tsRNA identified by (8) and the 20-nt upstream region of tRNA human genes for the 5′ leader RNAs. Human tRNA genes were taken from GtRNAdb (http://gtrnadb.ucsc.edu/) (40). Subsequently, we examined sncRNA-seq datasets of NCI-60 cell lines as provided by the sequence read archive (SRA) (PRJNA390643) (39), as well as sncRNA-seq datasets on TCGA. In Table 1, we provide a list of NCI-60 cell lines and the SRA datasets, while in Table 2, it lists the analyzed TCGA cancer types with their relative numbers of tumor and control samples. Raw FASTQ files were pre-processed for adaptor removal and quality filtering by applying Trim Galore (https://www.bioinformatics.babraham.ac.uk/projects/trim_galore/) tuned for sncRNA-seq (Phred quality score ≥20). Trim Galore is a wrapper for Cutadapt (41) and FastQC (http://www.bioinformatics.babraham.ac.uk/projects/fastqc/), which is used as a consistent method to apply quality filtering and adaptor trimming. Filtered FASTQ files were then aligned to a reference human genome (hg19) using TopHat version 2.1.0 (42) as well as to our custom annotation file. Read quantification has been performed with HTSeq version 0.10.0 (43). In this phase, all ambiguously mapped reads were removed for a more accurate and conservative analysis. Data analysis was performed with R version 3.5.1. Raw counts were normalized with two different normalization methods: transcripts per million mapped reads (TPM) (44) and reads per million mapped reads (RPM) (45).

$$ \mathrm{RPM}=\frac{\mathrm{number}\ \mathrm{of}\ \mathrm{reads}\ \mathrm{mapped}\ \mathrm{to}\ \mathrm{a}\ \mathrm{gene}\times {10}^6}{\mathrm{total}\ \mathrm{number}\ \mathrm{of}\ \mathrm{mapped}\ \mathrm{reads}\ \mathrm{for}\ \mathrm{a}\ \mathrm{given}\ \mathrm{library}} $$

$$ \begin{align*} \mathrm{TPM}&=\frac{\mathrm{number}\ \mathrm{of}\ \mathrm{reads}\ \mathrm{mapped}\ \mathrm{to}\ \mathrm{a}\ \mathrm{gene}}{\mathrm{gene}\ \mathrm{length}\ \mathrm{in}\ \mathrm{bp}}\\ &\quad \times \left(\frac{1}{\sum \frac{\mathrm{number}\ \mathrm{of}\ \mathrm{reads}\ \mathrm{mapped}\ \mathrm{to}\ \mathrm{a}\ \mathrm{gene}}{\mathrm{gene}\ \mathrm{length}\ \mathrm{in}\ \mathrm{bp}}}\right)\times {10}^6 \end{align*}$$

**Table 1:** List of NCI-60 cell lines tested and the relative SRA dataset

Cell line	Type of cancer	SRA dataset
T-47D	BREAST	SRR5689215
MCF-7	BREAST	SRR5689213
MDA-MB-231	BREAST	SRR5689212
BT-549	BREAST	SRR5689211
HS-578T	BREAST	SRR5689210
SF-295	CNS	SRR5689217
SF-268	CNS	SRR5689216
SF-539	CNS	SRR5689214
U251	CNS	SRR5689209
SNB-75	CNS	SRR5689208
SNB-19	CNS	SRR5689175
HCT-116	COLON	SRR5689179
HT-29	COLON	SRR5689178
KM12	COLON	SRR5689177
SW-620	COLON	SRR5689176
COLO 205	COLON	SRR5689174
HCT-15	COLON	SRR5689173
HCC2998	COLON	SRR5689172
MOLT-4	LEUKEMIA	SRR5689193
K-562	LEUKEMIA	SRR5689192
SR	LEUKEMIA	SRR5689191
RPMI 8226	LEUKEMIA	SRR5689190
CCRF-CEM	LEUKEMIA	SRR5689183
HL-60(TB)	LEUKEMIA	SRR5689182
LOX-IMVI	MELANOMA	SRR5689197
MALME-3M	MELANOMA	SRR5689196
MDA-MB-435	MELANOMA	SRR5689195
M14	MELANOMA	SRR5689194
SK-MEL-5	MELANOMA	SRR5689189
SK-MEL-28	MELANOMA	SRR5689188
UACC-62	MELANOMA	SRR5689167
UACC-257	MELANOMA	SRR5689164
SK-MEL-2	MELANOMA	SRR5689165
NCI-H522	NSCLC	SRR5689201
NCI-H460	NSCLC	SRR5689200
HOP 62	NSCLC	SRR5689171
NCI-H23	NSCLC	SRR5689170
EKVX	NSCLC	SRR5689169
HOP 92	NSCLC	SRR5689168
A549	NSCLC	SRR5689166
NCI-H322M	NSCLC	SRR5689163
NCI-H226	NSCLC	SRR5689162
OVCAR-5	OVARIAN	SRR5689207
OVCAR-4	OVARIAN	SRR5689206
OVCAR-8	OVARIAN	SRR5689205
OVCAR-3	OVARIAN	SRR5689204
NCI/ADR-RES	OVARIAN	SRR5689203
IGR-OV1	OVARIAN	SRR5689202
SK-OV-3	OVARIAN	SRR5689198
DU-145	PROSTATE	SRR5689199
PC-3	PROSTATE	SRR5689187
A498	RENAL	SRR5689186
CAKI-1	RENAL	SRR5689185
786-0	RENAL	SRR5689184
SN12C	RENAL	SRR5689181
TK-10	RENAL	SRR5689180
UO-31	RENAL	SRR5689161
ACHN	RENAL	SRR5689160
RXF393	RENAL	SRR5689159

**Table 2:** List of analyzed TCGA cancer types with their relative numbers of tumor and control samples

Tumor type	Tumor name	Tumor samples	Control samples
ACC	Adrenocortical carcinoma	79	
BLCA	Bladder urothelial carcinoma	408	19
BRCA	Breast invasive carcinoma	1101	113
CESC	Cervical squamous cell carcinoma and endocervical adenocarcinoma	306	3
CHOL	Cholangiocarcinoma	36	9
COAD	Colon adenocarcinoma	459	41
DLBC	Lymphoid neoplasm diffuse large B-cell lymphoma	48	
ESCA	Esophageal carcinoma	185	11
GBM	Glioblastoma multiforme	167	
HNSC	Head and neck squamous cell carcinoma	522	44
KICH	Kidney chromophobe	66	25
KIRC	Kidney renal clear cell carcinoma	534	72
KIRP	Kidney renal papillary cell carcinoma	291	32
LAML	Acute myeloid leukemia	173	
LGG	Brain lower grade glioma	533	
LIHC	Liver hepatocellular carcinoma	374	50
LUAD	Lung adenocarcinoma	517	59
LUSC	Lung squamous cell carcinoma	501	51
MESO	Mesothelioma	87	
OV	Ovarian serous cystadenocarcinoma	309	
PAAD	Pancreatic adenocarcinoma	179	4
PCPG	Pheochromocytoma and paraganglioma	184	3
PRAD	Prostate adenocarcinoma	498	52
READ	Rectum adenocarcinoma	166	10
SARC	Sarcoma	263	2
SKCM	Skin cutaneous melanoma	472	1
STAD	Stomach adenocarcinoma	414	35
TGCT	Testicular germ cell tumors	139	
THCA	Thyroid carcinoma	513	59
THYM	Thymoma	120	2
UCEC	Uterine corpus endometrial carcinoma	546	23
UCS	Uterine carcinosarcoma	57	
UVM	Uveal melanoma	80	
		**10 327**	**720**

**Figure 1:**
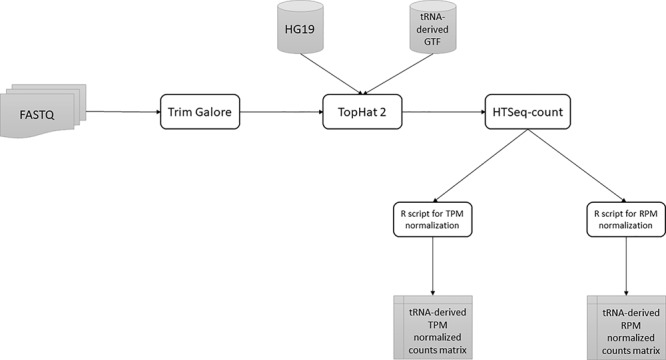
A summary of the tRNA-derived ncRNAs identification pipeline from RAW FASTQ files to normalized counts matrix.

All tsRNAs, 5′ leader RNAs and tRFs with average log2 TPM less than 1 were removed. A summary of our full pipeline is shown in Figure 1.

### Implementation of tRFexplorer

All identified tsRNAs, 5′ leader RNAs and tRFs with their expression profiles have been integrated in a novel database named tRFexplorer. tRFexplorer enables users to visualize the expression profile of each tRNA-derived ncRNA in both NCI-60 cell lines and TCGA samples (33 cancer types). Furthermore, it uses the R package limma (46) to perform DE analysis on TCGA data. Interactive visualization of its results has been implemented through the R package Glimma (47). Our database allows users to conduct correlation analysis of tRNA-derived ncRNAs expression in NCI-60 with all data available on CellMiner (48, 49). Correlation analysis with genes and miRNA expression profiles, as well as patient survival, of TCGA samples has also been implemented.

tRFexplorer was developed by employing PHP and R for its backend, and Javascript and React for the main user interface. All omics data and compound activities used for the correlation analysis were obtained from CellMiner (48, 49). In Table 3, we list all CellMiner datasets.

**Table 3:** A list of CellMiner datasets with their description

**CellMiner dataset**	**Description**
DNA CNV-Roche NibleGen 385 K aCGH	385 K element tiling array based on NCBI Build 35 of the human genome (HG17) and re-mapped to NCBI Build 35 (HG19); 50-mer tiling with a median probe spacing of 6000 bp.
DNA CNV-combined aCGH	Probe intensities combined from four platforms: agilent human genome CGH Microarray 44A, Nimblegen HG19 CGH 385 K WG Tiling v2.0, Affymetrix GeneChip human mapping 500 k array set and Illumina Human1Mv1_C Beadchip
DNA single nucleotide polymorphism (SNP) per gene-Affy 500 K	This platform is used for whole-genome association studies. It is comprised of two arrays which enable genotyping of more than 500 000 SNPs.
DNA SNP per gene-Illumina 1 M SNP	BeadChip array based on Illumina’s infinium assay with probes for 1 072 820 SNPs
DNA methylation-Illumina 450 K	Approximately 450 000 probes querying the methylation status of CpG sites within and outside of genes.
RNA Affy HG-U133_AB	Human genome U133. 44 000 probeset 2-chip set. Gene expression.
RNA Affy HG-U133 Plus 2.0	Aproximately 47 000 transcripts
RNA Affy HuEx 1.0	1 432 155 probesets for all human gene exons
RNA agilent human mRNAs	44 000 probes for approximately 41 000 genes, with four arrays spotted on each slide.
RNA expression combined z-scores	Gene expressions
RNA agilent human miRNAs	15 000 probes for 723 human and 76 human viral miRNA’s. Each slide contains eight arrays.
RNA microRNA OSU V3 Chip	Custom microarray developed at Microarray Shared Resource Comprehensive Cancer Center, OSU microarray facility. It contains 11 k probes (two technical replicates) for murin and human microRNAs together with hypothetical microRNAs and control probes.
RNA ABC transporters array	47 specific oligonucleotide probes were designed for each of the ABC transporters using DNAStar Primer Select. Expression levels were measured by real-time quantitative RT-PCR using the LightCycler RNA Amplification SYBR Green kit and a LightCycler machine.
RNA OSU transporter array	Spotted 70-mer microarray
Protein lysate array	Reverse-phase lysate arrays (RPLA) for 162 antibodies for 94 genes. Each array included 64 lysates (60 cancer cells and 4 replicate control pools) in 10 serial 2-fold dilutions.
Compound activities	Negative log10 (GI50) values of sulforhodamine B assay for ~ 50 K compounds, including more than 20 000 that passed quality control, 158 Food and Drug Administration approved and 79 clinical trial drugs. Higher values equate to higher sensitivity of cell lines.

Genomic viewer for tRNA-derived ncRNAs visualization is based on JBrowse (50). JBrowse is a fast and interactive genomic viewer built entirely with new HTML5 technology. We customized our browser by allowing users to search for both tRNAs or tRNA-derived ncRNAs using both genomic coordinates or identifiers.

## Results

### tRNA-derived ncRNAs identified

In our study, we employed NCI-60 (39) and TCGA sncRNA-seq datasets to identify tsRNAs, 5′ leader RNAs and tRFs, assessing their expression profile. In these datasets, we were able to identify 322 expressed tRNA-derived ncRNAs in NCI-60 (11 tRF-5s, 55 tRF-3s, 107 tsRNAs and 149 5′ leader RNAs) and 232 expressed tRNA-derived ncRNAs (53 tRF-5s, 58 tRF-3s, 63 tsRNAs and 58 5′ leader RNAs) in TCGA. A number of tsRNAs, 5′ leader RNAs and tRFs identified across NCI-60 cell lines and TCGA samples present noticeable expression levels. Moreover, all small RNA sequences mapped within four specific regions: 5′ end (tRF-5) and 3′ end (tRF-3) of mature tRNA, and 3′ trailer (tsRNA or tRF-1) and 5′ leader (5′ leader RNAs) regions of primary tRNA genes. If these small RNA sequences were the result of a random degradation process, their ends would be equally distributed along the lengths of tRNA genes with a comparable frequency (34, 35). In addition, we can observe that each TCGA cancer type (whose control samples are available) displays a different pattern of dysregulated tRNA-derived ncRNAs. Taken together, these results may suggest that these small RNAs are not fragments derived from the random cleavage of precursor and mature tRNAs, rather they are actively expressed and produced by specific ribonucleases and may be dysregulated in several human cancers. Indeed, recent evidences have shown dysregulated tRNA-derived ncRNAs in Chronic lymphocytic leukemia (CLL), colon, breast, ovary, lung and prostate cancers (8, 9, 11, 12, 51, 52).

### tRFexplorer database

All identified sncRNAs have been integrated in a novel database named tRFexplorer. tRFexplorer is an easy-to-use, web-based database (https://trfexplorer.cloud/) containing tRNA-derived ncRNAs expression profiles for NCI-60 cell lines and TCGA samples, together with all omics and compound activities data available on CellMiner (Table 3). Leveraging CellMiner data, tRFexplorer enables users to perform correlation analysis inferring knowledge on the biological function of such molecules. Furthermore, a module allowing DE analysis for all tRNA-derived ncRNAs in TCGA samples has been released. A detailed explanation of tRFexplorer functions is provided in the following sections.

**Figure 2:**
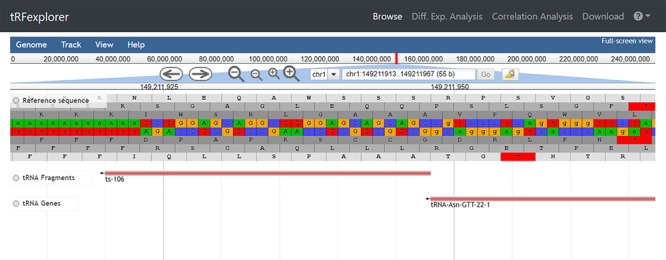
The genomic location of ts-106 visualized in our interactive genome viewer.

*Browse.* In the ‘Browse’ section, users can search for tsRNAs, 5′ leader RNAs and tRFs by ‘location’ or ‘expression’. Browsing by location enables users to search and visualize all tRNA-derived ncRNAs in the reference human genome (Figure 2). Specifically, through the custom genome browser, it is possible to interactively search tRNA-derived ncRNAs either by genomic coordinates or by identifier.

Browsing by expression section enables users to filter data using at least one of the following options: (i) the type of fragment (tRF-3, tRF-5, tsRNA and 5′ leader RNAs); (ii) the amino acid carried by the pre-tRNA; (iii) the anticodon sequence; (iv) the dataset in which the fragment is expressed (TCGA tumor types or NCI-60 cell lines); and (v) the tissue subtype (normal, tumor, metastatic, recurrent, etc). It is also possible to set a minimum RPM threshold for tRNA-derived ncRNAs. The search procedure will scan our database looking for all tRNA-derived ncRNAs matching users criteria, and the results will be reported in a table.

**Figure 3:**
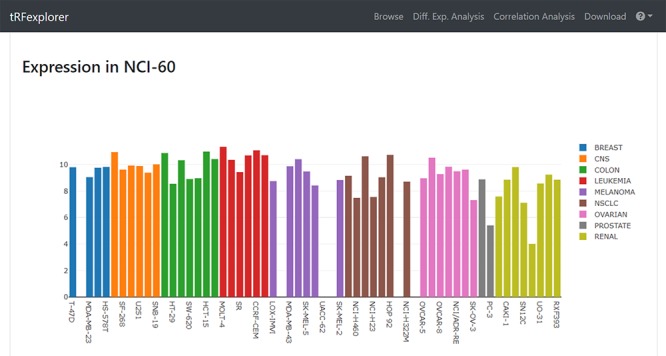
Example of bar plot which shows tRFdb-3022a expression across NCI-60 cell line.

**Figure 4:**
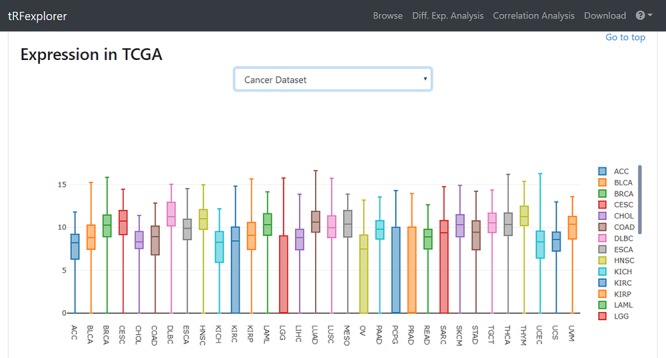
Example of box plot which shows tRFdb-3022a expression across TCGA cancer types.

Once results become available, users may view a page with detailed information by selecting any single result. Such page will show plots for assessing RPM expression levels in both NCI-60 (Figure 3) and TCGA (Figure 4). A genomic viewer will show genomic locations for each tRNA-derived fragment.

**Figure 5:**
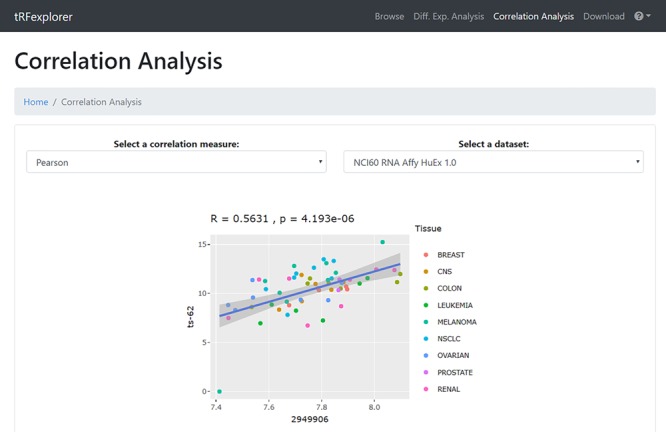
Example of a scatter plot which shows the correlation between the expression profile of ts-62 in NCI-60 and the expression of NOTCH4.

*Correlation analysis.* In the ‘Correlation Analysis’ section, users can perform correlation analyses of all identified tsRNAs, 5′ leader RNAs and tRFs in NCI-60, with the omics and compound activities data available on CellMiner (48) (Table 3). Correlation analysis can also be performed with mRNA/miRNA expression profiles, as well as patient survival data, of TCGA samples. Specifically, the user selects the correlation measure (Pearson or Spearman) and which dataset to consider. A list of correlated and anticorrelated tRNA-derived ncRNAs will be shown. The results can then be filtered by: (i) ncRNA name; (ii) genes, miRNAs, compound names; (iii) the genomic coordinates, when available; and (iv) the minimum correlation value. By clicking on each result, an interactive scatter plot with the data of the selected molecules will appear (Figure 5).

**Figure 6:**
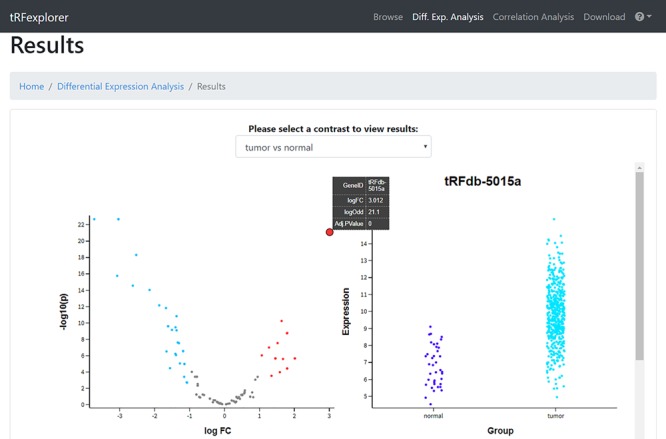
Vulcano plot which shows the differentially expressed tRNA-derived ncRNAs in TCGA Lung Squamous Cell Carcinoma (LUSC) when compared with control samples, and a swarm plot which specifically shows the difference in the expression profile of one (tRFdb-5015a) of the differentially expressed tRNA-derived ncRNAs.

*DE analysis.* In the ‘DE analysis’ section, users can perform DE analysis to discover which tsRNAs, 5′ leader RNAs and tRFs are dysregulated in TCGA tumor types. To start the analysis, users select the cancer type and one of the available covariates (gender, race, vital status, sample type or classification). It is also possible to set the maximum *P*-value and minimum log-fold-change (logFC) for the analysis. After selecting all the parameters, users must select at least one contrast to perform for the DE analysis, in association with the selected covariate. Once the analysis is launched, a list of differentially expressed tRNA-derived ncRNAs with their logFC and FDR adjusted *P*-value will be shown together with an interactive volcano plot to better visualize their DE (Figure 6). By clicking on a specific point in the plot or row in the table, a swarm plot of the expression values will also be shown (Figure 6).

### Comparison between tRFexplorer and MINTbase V.2.0

We compared data and features of tRFexplorer with MINTbase V2.0 (53), another established database of tRNA-derived ncRNAs in TCGA. MINTbase V2.0 has a user-friendly graphical user interface and it combines data from 768 different human datasets and 11.198 TCGA small RNA-Seq datasets. MINTbase V2.0, however, is focused only on tRFs, i-tRF and tiRNAs (or tRNA halves). Indeed, only tRNA-derived ncRNAs formed from mature tRNAs are taken into account. tsRNAs and 5′ leader RNAs, coming from pre-tRNAs, are not available in MINTbase V.2.0. On the other hand, tRFexplorer does not store tiRNAs and i-tRF. Table 4 reports the tRNA-derived ncRNAs classes stored in tRFexplorer and MINTbase V.2.0, respectively.

Concerning tRNA-derived ncRNAs identification methods, we aim to stress an important difference between tRFexplorer and MINTbase. The tRFs available in MINTbase V2.0 have been identified with MINTmap (54) prediction algorithm. Instead, tRFexplorer uses well-known mapping and counting tools (see Materials and Methods 2.1) to identify the RNA reads mapping on our custom annotated human genome. Specifically, we used the genomic coordinates obtained from tRFdb (34) for the identification of tRF-5s and tRF-3s and the genomic coordinates published on (8) for the identification of the experimental validated tsRNAs. Finally, for the 5′ leader RNAs identification, we counted the RNA reads mapped on the 20-nt upstream regions of all human tRNA genes. We used this strategy since tRFdb nomenclature is the most widely used for tRFs in literature while we used the genomic coordinates present on (8) for tsRNAs since they were identified by *in vitro* experiments and not with in-silico prediction.

Moreover, one of the aims of our work was to report only high confident tRFs and 5′ leader RNAs and *in vitro* validated tsRNAs. Specifically, we reported 399 tRNA-derived ncRNAs on tRFexplorer (some of them are expressed both on NCI-60 and TCGA datasets, while others are specifically expressed either on NCI-60 or TCGA datasets). On the contrary, a very large number of unique sequences reported on MINTbase V.2.0 (28.824) were identified through the prediction algorithm MINTmap. We noticed that many tRFs, i-tRFs and tiRNAs reported in MINTbase V.2.0 are expressed with 1 RPM in a single dataset (1 out of 12.023). Specifically, 6.248 out of 28.824 unique sequences present on MINTbase V.2.0 are expressed in only one dataset (1 out of 12.023), while 15.717 out of 28.824 unique sequences are expressed in only 10 out of 12.023 datasets. It is also well established that tRF-5s and tRF-3s have well-defined length and they can be further stratified in tRF-5a (14–16 nts), tRF-5b (22–24 nts), tRF-5c (28–30 nts), tRF-3a (about 18 nts) and tRF-3b (about 22 nts) subclasses accordingly to their size (13, 15, 30, 32). Moreover, tRF-5s and tRF-3s are produced by ribonuclease cleavage in the D-loop and T-loop region, respectively (13, 15, 30, 32). In MINTbase V2.0, we noticed that 252 out of 2304 tRF-5s and 1439 out of 4028 tRF-3s are longer than 31 and 25 nt, respectively, while 248 out of 2304 tRF-5s and 284 out of 4028 tRF-3s have sequences that do not end at their cleavage site in the D-loop or T-loop, respectively, but they overlap the anticodon region.

**Table 4:** tRNA-derived ncRNAs classes stored in tRFexplorer and MINTbase V2.0

	tRFexplorer	MINTbase V.2.0
5′ Leader RNAs	x	
tsRNAs	x	
tRF (tRF-5s and tRF-3s)	x	x
tiRNAs (tiRNA-5s and tiRNA-3s)		x
i-tRF		x

Indeed, the total number of tRNAs-derived ncRNAs in MINTbase V2.0 looks quite high if we consider that in the human genome, there are only about 400 tRNA genes and that many 5′ and 3′ end regions of mature tRNAs are shared across different tRNAs, and therefore, a single tRFs, with the same sequence, can be produced by different tRNAs. For this reason, to avoid overestimation, we decided to keep very low the number of identified tRNA-derived ncRNAs.

Finally, we conducted a comparison between the features of tRFexplorer vs those available in MINTbase V.2.0. tRFexplorer implements several specific functions such as: explore the genomic location of tRNA-derived ncRNAs through an interactive genomic browser; perform DE analysis in all TCGA samples; and finally correlation analysis with different TCGA (mRNAs and miRNAs expression) and CellMiner (omics data and compounds activities) data. Moreover, different high-quality plots are available on tRFexplorer to better understand the results. A detailed comparison of the features is shown in Table 5.

**Table 5:** Features comparison of tRFexplorer and MINTbase V2.0

	tRFexplorer	MINTbase V.2.0
Datasets	TCGA, NCI-60	TCGA, human datasets
Total number datasets	12.187 + 59 = 12.246	12.023
tRNA-derived ncRNAs identification methods	Mapping and counting on custom annotated human genome	Prediction based on MINTmap
tRNA-derived ncRNAs filtering expression criteria	Mean Log2 TPM > 1 across all samples	RPM ≥ 1 in a single sample
Unique sequences	399	28.824
tRFs sub-classification according to their size	Available	Not available
Normalization methods	TPM, RPM	RPM
Interactive genomic browser	Available	Not available
DE analysis	Available	Not available
Correlation analysis	Available	Not available
Plot for data visualization	Bar plot, box plot, scatter plot, volcano plot, swarm plot	Bar plot, box plot
Other data for tRNA-derived ncRNAs correlation analysis	TCGA mRNAs and miRNAs expression, CellMiner datasets	No other data available

## Discussion

Our study aims at identifying tsRNAs, 5′ leader RNAs and tRFs in NCI-60 and TCGA small RNA-seq datasets, together with an intuitive web interface for data querying and browsing. For this purpose, we implemented a custom bioinformatics pipeline for the detection and quantification of these ncRNAs from small RNA-Seq data. Due to TCGA’s large sample base, we took into consideration NCI-60 in light of its extensive use for the testing of novel compounds and the identification of drug-response biomarkers (55–60). We analyzed recent versions of the NCI-60 (39) and TCGA small RNA-seq datasets to identify and establish expression profiles for tsRNAs, 5′ leader RNAs and tRFs. In these datasets, we were able to detect 322 expressed tRNA-derived ncRNAs in NCI-60 (11 tRF-5s, 55 tRF-3s, 107 tsRNAs and 149 5′ leader RNAs) and 232 expressed tRNA-derived ncRNAs in TCGA samples (53 tRF-5s, 58 tRF-3s, 63 tsRNAs and 58 5′ leader RNAs). Our reference tsRNA and tRF annotation were obtained from (8) and tRFdb (34), respectively. 5′ leader RNAs were obtained from the 20-nt upstream region of tRNA genes and named by using the tRNA identifier from which they derived.

It is noteworthy that the high expression levels of some tsRNAs, 5′ leader RNAs and tRFs in both NCI-60 and TCGA, and their DEs across TCGA cancer types indicate that these molecules are not fragments derived from the random cleavage of precursor and mature tRNAs, suggesting that such molecules may be actively expressed and produced by specific ribonucleases. Indeed, if tsRNAs, 5′ leader RNAs and tRFs were the results of a random degradation process, their ends would be equally distributed along the lengths of tRNA genes with a comparable frequency (34, 35). In our case, small RNA sequences mapped on four specific regions: 5′ end (tRF-5) and 3′ end (tRF-3) of mature tRNA, and 3′ trailer (tsRNA or tRF-1) and 5′ leader (5′ leader RNAs) regions of primary tRNA genes. Pre-tRNA molecules undergo a maturation process inside the nucleus, where 5′ leader and 3′ trailer sequences are cleaved by RNAse P and RNAse Z, respectively (15). It is established that tsRNAs are derived from the 3′ trailer region of pre-tRNAs (9). Conversely, the identification of small RNA sequencing reads mapping within the pre-tRNA 5′ leader genomic region represents an additional evidence of this yet poorly investigated class of tsRNAs (24, 25, 61). Although some authors already reported tRNA fragments mapping between 5′ leader region and mature tRNA (36, 62), our 5′ sequences are entirely located inside the pre-tRNA leader region. Furthermore, such sequences extend up to the RNAse P cleavage site, without going beyond such site. The high expression of 5′ leader RNAs and their specific ends in correspondence of RNAse P cutting site supports the hypothesis that these molecules could have a biological function, although an *in vitro* validation is still missing.

All identified tsRNAs, 5′ leader RNAs and tRFs have been stored in tRFexplorer, a novel database accessible through an easy-to-use web interface. tRFexplorer allows us to visualize the expression profile of each tsRNAs, 5′ leader RNAs and tRFs for all NCI-60 cell lines and TCGA tumor types. Furthermore, it enables DE analysis for all tumor types present in TCGA and correlation analysis with all omics and compounds activity data reported on CellMiner. The interface is comprised of three main sections: ‘Browse’; ‘DE Analysis’; and ‘Correlation Analysis’. The ‘Browse’ section is used to search tsRNAs, 5′ leader RNAs and tRFs by location and expression. For each sncRNA, its expression is displayed in both NCI-60 cell lines and TCGA tumor types. Moreover, an interactive genomic viewer allows users to search and visualize all the identified tRNA-derived ncRNAs in the reference human genome. Through the DE analysis panel, users can discover which tRNA-derived ncRNAs are dysregulated in each TCGA tumor type. By selecting the cancer type to analyze and one of their variables (gender, race, vital status, sample type or classification), together with the *P*-value threshold and minimum logFC, a list of differentially expressed tRNA-derived ncRNAs with their logFC and FDR adjusted *P*-value will be shown. Volcano plots and swarm plots are also shown in order to better elucidate their DE. Finally, through the ‘Correlation Analysis’ panel, the user can perform correlation analyses of all identified tRNA-derived ncRNAs in NCI-60 with the omics and compound activities data available on CellMiner (48) (Table 3). In addition, correlation analysis with genes, miRNA expression and patients’ survival data of TCGA samples are also possible. The correlation tool will yield only the molecules that have an absolute correlation coefficient greater than 0.5. The user also has the option to select two different correlation measures (Pearson and Spearman).

The correlation analysis aids in the investigation of the biological function of tRNA-derived ncRNAs, as there is currently poor evidence in such regard. For this purpose, their correlation or anticorrelation with protein levels, mRNA and miRNA expressions, could provide a first clue regarding a potential biological pathway in which these ncRNAs may be involved.

Moreover, tRNA-derived ncRNAs showing high correlations or anticorrelations with the activity profiles (chemoresistance and chemosensitivity) of a specific antitumor compound could be selected for further studies as drug response biomarkers. Indeed, in a recent study, it has been shown that some tRFs are dysregulated in cell lines of breast cancer resistant to Trastuzumab against sensitive cell lines (63).

Finally, we compared data and features of tRFexplorer with MINTbase V2.0 (53), a database of tRNA-derived ncRNAs in TCGA. From the comparison, we can establish that, to avoid overestimation and artifacts within the databases, tRFexplorer stores less tRNA-derived ncRNAs with respect to MINTbase. On the other hand, tRFexplorer presents a more flexible interface allowing rich functional data analysis.

## Conclusion

This study introduces a novel database called tRFexplorer equipped with a Web App enabling the exploration of tRNA-derived ncRNAs expression profiles (tsRNAs, 5′ leader pre-tRNAs derived small RNAs, and tRFs) on the NCI-60 panel and TCGA samples. By exploiting DE analysis, tRFexplorer allows users to study the specific pattern of dysregulated tRNA-derived ncRNAs of each TCGA cancer type. Finally, a correlation analysis tool provided by our software may be employed as an instrument to investigate the putative biological roles of these molecules in the absence of direct experimental validation of their functions while allowing users to consider further *in vitro* and *in vivo* investigations on the most promising molecules.

## Availability

tRFexplorer is available at https://trfexplorer.cloud/.
